# Intonation words in initial intentional communication of Mandarin-speaking children

**DOI:** 10.3389/fpsyg.2024.1366903

**Published:** 2024-05-28

**Authors:** Yunqiu Zhang, Jiantao Li, Yang Zhang

**Affiliations:** ^1^Language Acquisition Laboratory, School of Literature, Capital Normal University, Beijing, China; ^2^Faculty of Linguistic Sciences, Beijing Language and Culture University, Beijing, China

**Keywords:** Mandarin-speaking children, before the age of 17 months, intentional communication, intonation words, initial acquisition of syntax

## Abstract

Intonation words play a very important role in early childhood language development and serve as a crucial entry point for studying children’s language acquisition. Utilizing a natural conversation corpus, this paper thoroughly examines the intentional communication scenes of five Mandarin-speaking children before the age of 1;05 (17 months). We found that children produced a limited yet high-frequency set of intonation words such as “啊 [a], 哎 [æ], 欸 [ε], 嗯 [ən], 呃 [ə], eng [əŋ], 哦 [o], and 咦 [i].” These intonation words do not express the children’s emotional attitudes toward propositions or events; rather, they are utilized within the frameworks of imperative, declarative, and interrogative intents. The children employ non-verbal, multimodal means such as pointing, gesturing, and facial expressions to actively convey or receive commands, provide or receive information, and inquire or respond. The data suggests that the function of intonation words is essentially equivalent to holophrases, indicating the initial stage of syntactic acquisition, which is a milestone in early syntactic development. Based on the cross-linguistic universality of intonation word acquisition and its inherited relationship with pre-linguistic intentional vocalizations, this paper proposes that children’s syntax is initiated by the prosodic features of intonation. The paper also contends that intonation words, as the initial form of human vocal language in individual development, naturally extend from early babbling, emotional vocalizations, or sound expressions for changing intentions. They do not originate from spontaneous gesturing, which seems to have no necessary evolutionary relationship with the body postures that chimpanzees use to change intentions, as suggested by existing research. Human vocal language and non-verbal multimodal means are two parallel and non-contradictory forms of communication, with no apparent evidence of the former inheriting from the latter.

## Introduction

Dascal and Berenstein (1987) pointed out that in the ‘original’ situation of communication between baby and caregiver, the term ‘duty’ may appear questionable. However, in a certain context, it holds justification. The baby’s complete dependency on parental assistance for survival, owing to its motor and psychological vulnerabilities, necessitates that caregivers interpret and comprehend the signals emitted by the baby as meaningful. This understanding, which imposes meanings onto the baby, often assumes a level of transparency. Despite this, both the baby and the caregivers eventually become aware of the opacity that characterizes their communication. Therefore, understanding perpetually strives to emulate the transparency and immediacy of the original symbiotic relationship, albeit with inherent limitations. In simpler terms, at this early stage, ‘grasping’ the baby’s communicative intention means understanding and acknowledging that the baby, within its limitations, can express its own needs. However, if the caregiver believes that understanding the baby’s communicative intention involves imposing his/her own intention onto the baby and ignoring the baby’s intention, then there’s a failure to understand. Essentially, the caregiver needs to recognize and understand the baby’s communicative intention, rather than projecting her own intention onto the baby. Furthermore, the baby does not see in the caregiver an autonomous source of intentions, nor a being who is able to either realize or not its (the baby’s) intention. The caregiver is seen just as an instrument, which has the unconditional ‘duty’ of realizing those intentions (Dascal and Berenstein, 1987). Hence, it can be understood that the communication intentions of young children, especially before 18 months, are not easy to interpret.

Some research underscored the significance of contextual clues in interpreting children’s intentions. Contextual cues, both verbal and nonverbal, play a crucial role in understanding what children mean and intend to communicate, particularly in early childhood when their language skills are still developing. By considering the situational context, caregivers and educators can more accurately decipher the meaning behind a child’s words or actions, leading to more effective communication and interaction with the child (Kachel et al., 2021; Lee and Lew-Williams, 2022).

Human communication is inherently multimodal and requires the integration of different types of cues. Nonverbal cues such as gestures and facial expressions play an important role in children’s prelinguistic communication (Tomasello, 2003, 2008; Colonnesi et al., 2010; Bohn and Köymen, 2018). Therefore, interpreting children’s communication intentions requires considering all contextual clues, while also examining explicit nonverbal and verbal cues.

Our observation of various pre-lexical life scenarios of children’s single-word utterances shows that young children employ two modes of expression: vocalization and gestural actions (including ocular communication). However, these various vocalizations and gestural actions are not intrinsically homogeneous. As language communication always involves entities beyond the communicating parties, forming a triadic relationship consisting of the communicating parties and the referred entities or events, only when children establish such a triadic relationship through vocalization or gestural actions as described earlier can it be considered a form of communication close to language, i.e., intentional communication. This type of communication is grounded in a cognitive skill called joint attention (Tomasello, 2012), which typically develops in infants between 9 to 12 months. Thus, only communicative acts initiated by infants aged 9 to 12 months such as pointing to objects and gesturing, accompanied by vocalizations like “嗯 [ən]” and “啊 [a]” involve referred entities or events. Such communicative acts exhibit clear intents in specific contexts, wherein adults can provide targeted responses or offer corresponding feedback, demonstrating a certain level of homogeneity with adult language communication. Other vocalizations such as babbling emerging as early as 3 to 4 months, changes in vocalization expressing altered intentions when desires are met or unmet (e.g., eager sounds when desiring milk), or emotional vocalizations, along with early gestures like waving arms appearing within 1 or 2 months after birth, as well as actions with intention-altering functions (such as extending both hands to be picked up or twisting the body to resist being held, sometimes accompanied by arching backward or crying), are not instances of intentional communication and do not fall within the scope of this study.

Regarding pre-linguistic intentional communication in infants, scholars predominantly focus on children’s gestural actions. However, it is observed that children do not solely rely on gestural actions for intentional communication. When expressing intentions through pointing, gesturing, and eye movements, children often accompany these actions with vocalizations like “嗯 [ən]” and “啊 [a].” Upon closer examination of communication scenes between children and others, it is noted that these accompanying vocalizations of “嗯 [ən]” and “啊 [a].” are almost redundant in terms of expressive function compared to pointing, gesturing, and facial expressions. In other words, they are generally consistent with the intention conveyed through pointing, gestures, facial expressions, and eye contact. The expressive function of the former, however, requires interpretation with the assistance of the latter. So, why do children make these “嗯 [ən],” “啊 [a]” sounds? It is also observed that as sporadic single-word utterances appear, children often use those utterances instead of the “嗯 [ən],” “啊 [a]” sounds. Based on those observations, we speculate that these accompanying “嗯 [ən],” and “啊 [a]” sounds are likely holophrases—single words that children want to say but cannot articulate. Some studies also discussed this related issue, such as Snow (2001, 2006), Snow and Balog (2002), and Snow and Ertmer (2009).

Although these “嗯 [ən]” and “啊 [a]” sounds when observed in isolation seem to lack concrete lexical meaning, they express clear communicative intent within the context, which we may refer to as “intonation words.” In summary, intonation words are phrasal substitutes that accompany infants and young children’s attempts to convey intentions through pointing, actions, or facial expressions, sharing a similar communicative function. Unlike early babbling and many early emotional vocalizations, intonation words always accompany pointing, gestures, and facial expressions and have clear syllabic boundaries and distinct intonation features. A rising tone is often used for questioning, while flat or falling tones are used for statements or imperatives. Let us illustrate this feature with examples.

Subject JBS was dancing with a mobile phone which had music on. After a few seconds, the music suddenly stopped. JBS looked up at Adult LXF crouching in front of him and made a “啊 [a]” sound with a rising tone, seemingly asking why there was no more music. LXF did not answer why but said, “jump, jump!” The mobile phone music resumed, and JBS jumped twice again. Then, she pointed at the phone screen with her index finger and made an “啊 [a]” sound with a falling tone, seemingly telling LXF that the music had returned. (JBS 1;05.12).

We used Praat software to conduct acoustic analysis on the pitch patterns of the two “啊 [a]” sounds produced by the child, and the spectrogram is presented below:

The left side of the spectrogram depicts the first rising “啊 [a]” produced by JBS, while the right side illustrates the second falling “啊 [a].” Despite a considerable difference in duration between the two “啊 [a]” sounds, it is not a decisive factor for determining the pitch pattern and thus can be disregarded. By examining the rising slope (Hz) in the first spectrogram and the descending slope (Hz) at the end of the second spectrogram, we can observe a certain correlation between the pitch pattern and communicative intent.^1^

Researchers generally agree that intonation is a component of early language development. Before children produce their first words, they have already mastered most or all of the intonation system (Locke, 1983). Many scholars have found that some children, in the late babbling stage, use intonation similar to that of adults (Dore, 1975; Crystal, 1986), describing this phenomenon as “jargon intonation” or “pre-lexical intonation” (equivalent to intonation word) (Peters, 1977).

In summary, the intonation words and accompanying non-verbal units are the content that this article aims to examine. Meanwhile the question of how young children engage in intentional communication with others at the onset of linguistic development is a worthwhile inquiry. Its value lies not only in portraying the communicative scenes of children during the emergence of language competence but also in exploring crucial questions related to syntactic development through the communicative modalities and acquisition data of children. For instance, do children possess syntactic knowledge at the initial stage of intentional communication? How does syntactic initiation occur? Additionally, it allows for an exploration of the origins of children’s vocal communication and whether there is an evolutionary relationship between human vocal language and primate gestural communication.

### Research objectives

The primary questions of interest include: Does the initial production of intonation words signify the beginning of children’s intentional communication? If so, what intentions can children express using intonation words? What cues do we rely on to interpret various communicative intentions of children? Additionally, building upon the findings of the research objectives, how to further explore the functional features of children’s production of intonation words? What does the acquisition of intonation words indicate?

### Current studies

Numerous scholars, both domestically and internationally, have noted the early vocalizations of “嗯 [ən]” and “啊 [a]” in children. In language acquisition studies on Mandarin-speaking children, researchers such as Li (2004), Liu (2009), Guo (2016), Peng (2016), and Wang (2018) found that children produce words with communicative functions even before the holophrastic stage, such as “啊 [a], 咦 [i], 嗯 [ən], eng [əŋ], 呃 [ə], 哦 [o], and哎 [æ].” Yuming Li referred to them as “language elements” (2004:46–55), while most other scholars termed them interjections (Liu, 2009; Guo, 2016; Peng, 2016), and some referred to them as emotional intonation words (Zhang and Chao, 2019). Similarly, foreign researchers such as Bornstein et al. (2004) and Soderstrom et al. (2008) found that English-speaking children produce interjections like “ah,” “oh,” and “eh” during the holophrastic stage. Dejima et al. (2009) discovered that two Japanese children, during the holophrastic and even the babbling stage, used rising and falling intonations to express questions, statements, and exclamations. Asano (1997) also studied the acquisition of three difficult-to-pronounce interjections in English-speaking children aged 11 months to 2;11 years. The main findings indicated that the participant children started producing the first interjection, “oops,” at 1;07 years with a total of 11 instances. The order of acquisition was “oops” first, followed by “yuck,” and lastly “ouch.” By 1;00 year, the participant children already understood all these interjections, but production showed a lag, primarily due to the difficulty in pronouncing the interjections. Stange (2009) also argued that pronunciation limitations caused a delay in the production of certain interjections by children. However, Asano (1997) and Stange (2009) focused on true interjections, which differ from what other scholars term as “interjections,” and are not of the same nature as the intonation words discussed in this paper.

Hu (2016) proposed that pre-linguistic vocalizations play a significant role in the construction of human syntax, linking vocalizations such as [əŋ], [i:], and [a] to the CP system in a bidirectional growth pattern of syntax, expressing children’s emotions and attitudes, and contributing to the construction of the CP layer. Hu’s (2016) concept of vocalizations is broader than the scope of intonation words in this paper, encompassing emotional vocalizations mentioned earlier and extending into intonation words used in intentional communication. Emotional vocalizations are produced during babbling, and after entering intentional communication, there are occasional instances of such vocalizations. While emotional vocalizations express children’s emotions and attitudes, such as excitement, satisfaction, urgency, displeasure, refusal, and more,^2^ after entering intentional communication, the intonational words produced by children do not express attitudes toward propositions or events. Instead, they rely on specific communicative scenarios during vocalization, along with contextual elements such as pointing, gestures, and facial expressions, to convey events or propositions. Therefore, in this paper, we separate from the CP the vocalizations such as “嗯[ən]” and “啊 [a]” produced by children after entering the intentional communication stage, instead of considering them as emotional vocalizations.

On the other hand, Braine (1963, 1971) observed that individual words gradually assumed the communicative functions of entire phrases. For example, a child’s utterance “dada” could convey meanings such as “Where is daddy?” or “I want daddy,” depending on the context. Braine termed these utterances as holophrastic or one-word expressions. Moreover, a holophrase requires context beyond the single word to be understood. The book “The Development of Children” emphasizes the significance of body language in the effective use and interpretation of holophrases. According to Lightfoot et al. (2008), “The single word, along with accompanying gestures and facial expressions, functions as the equivalent of a complete sentence. Therefore, the single word itself is not a holophrase but rather a component within a broader framework of communication that encompasses nonverbal behaviors.” Based on this, it can be inferred that the examination of intonation words cannot be separated from the examination of non-verbal units.

In summary, existing studies, while acknowledging intonation words and their communicative functions, have not generally treated them as linguistic components. Even Radford (1990) does not consider English children’s production of “ah,” “oh,” “eh,” etc. as linguistic components. Consequently, there has been insufficient independent examination and effective discussion of vocalization phenomena occurring in such communicative contexts. This article aims to conduct a meticulous observation of intentional communication scenes in children before the age of 1;05, describing the communicative intentions conveyed by intonation words and interpreting contextual clues. Simultaneously, it depicts the usage of non-verbal units that are inseparable from intonation words. Based on this foundation, the article delves into early syntactic development issues in children.

## Method

### Participants

This study is based on a corpus of multiple case studies, and the participants were selected from the Speech Acquisition Laboratory at Capital Normal University. We utilized naturalistic language samples from five children before the age of 1;05, including YZR and YZX, who are twin boys. Relevant information about the participant children is presented in Table 1.

**Table 1:** Information table for five participant children’s language samples.

Child	Gender	Age range	Corpus duration (hours)	Residence	Family language
JBS	Female	1;02–1;05	16	Beijing	Mandarin
LCY	Male	0;10–1;05	23	Beijing	Mandarin
ZRY	Female	0;10–1;04	20	Beijing	Mandarin
YZR	Male	1;00–1;05	19	Beijing	Mandarin
YZX	Male	1;00–1;05	19	Beijing	Mandarin

### Procedure

We established long-term collaboration with each child’s parents, visiting the children’s homes every week to conduct one-hour audio and video recordings. The video recordings captured natural interactions during the children’s daily communication or play sessions with experimenters or parents, free from any external intervention.

Although the language data of the participant children were annotated using the CHAT format within the CHILDES system (MacWhinney, 2000), our research goals led us to eschew retrieval through the CLAN program. Instead, we manually transcribed communication scenes containing intonation particles, the acoustic features of intonation particles, as well as gestures, pointing, and facial expressions observed in the videos. Furthermore, representing the intonation particles using Chinese characters is not entirely precise, and the same applies to the use of International Phonetic Alphabet (IPA) annotations in some cases. For instance, the pronunciation of “啊 [a]” by children does not exhibit the same wide opening as in adults and sometimes resembles “哎 [æ].” Although both should be rendered as “啊 [a],” we account for the differences in tongue movement capabilities between children and adults, designating instances with a lower tongue position and larger mouth opening as “啊 [a]” and those with a slightly higher tongue position and a slightly smaller mouth opening as “哎/欸 [æ].” As for the child-produced “[əŋ],” we cannot find a corresponding Chinese character and tentatively transcribe it as “eng” using pinyin.

Moreover, intonational words exhibit different pronunciation characteristics. [ɑ/æ/ε] are open front vowels, with the tongue position between half-close and close, all unrounded; [ǝn/ǝ/ǝŋ] are mid-central vowels and the tongue position for each vowel is between half-close and close; [i] is a close front unrounded vowel; [oʊ] is a close-mid back rounded diphthong, with a low tongue position. According to the different pronunciation characteristics, we categorize intonational words into four types.

## Measure

### Based on what can one argue that prosodic words serve the function of intentional communication?

We may confirm the communicative intent of intonation words based on two reasons: first, intonation words align closely with the expression of intent through gestures, pointing, and facial expressions, the difference being the latter serve as cues for interpreting the intent of the former. They do not express emotional attitudes toward the events or propositions conveyed through gestures. Second, as children transition to the later stages of the age range examined in our corpus and begin to make holophrastic utterances, they occasionally replace intonation words with holophrases in similar communicative contexts, as illustrated in examples (2) and (3):

In a scene where our research assistant LYI responsible for sampling sat with the participant child JBS on the couch eating oranges, after JBS finished eating, JBS ran toward her mom holding the orange peel. LYI said, “Hey, come back, come back.” JBS handed the orange peel to her mom and exclaimed, “嗯 [ən]! 嗯 [ən]!” The mom asked, “嗯 [ən] what? Say it: 给 [kε55] (give).” JBS said, “给 [kε55].” The mom acknowledged JBS, saying, “Oh, right, say it again.” (JBS 1;03.07).

In this scene, the sampling research assistant TWW and the participant child LCY were playing with a toy car on the floor. After a while, TWW said, “Pipi, let us read. Let us see where your book is.” LCY said, “不 [pǝ55] (no),” shaking his head and looking at TWW. TWW continued, “啊 [a], do not want to read? Read it!” LCY shook his head again, uttering “欸 [æ].” (LCY 1;02.29).

### How to classify and quantify the functions of intonation words?

The confirmation and interpretation of different interactive contexts in this article are conducted based on intonation words as clues. Confirming and quantifying intonation words also represents the confirmation and interpretation of contexts. While intonation words admittedly have communicative intent, their specific intent requires interpretation through the communicative context and the elements of gestures, pointing, and facial expressions, namely, the interactive contextual interpretative elements. We refer to these elements collectively as the interpretative elements of the interactive context. A detailed analysis of these interpretative elements is necessary to systematically depict the communicative intent expressed by intonation words.

The interpretative elements can be further examined from the aspects of nonverbal multimodal means (such as pointing, gestures, and facial expressions), communicative roles, and intent frameworks. Intent frameworks include declarative, interrogative, and imperative intents, with each intent framework further dividing the communicative roles of children into informants and recipients, questioners and answerers, and command-givers and command-receivers. Nonverbal multimodal means can be categorized into three types: (1) action, such as in Examples (4), the child’s action of patting the rocking horse in this communicative scenario constitutes a gesture, and later reaching out to the sampling research assistant also qualifies as an action. (2) Expression. The child patted the rocking horse while at the same time looking up at his mother. The act of looking at the mother constitutes facial expression, also referred to as eye contact. Subsequently, when the child looked at the sampling research assistant, it also falls under facial expression. (3) Pointing. In Example (5), the child pointing outside of the window with his index finger is the use of pointing as a non-verbal communicative element.

1. In a scenario where the participant child YZR wanted to climb onto a rocking horse but failed, YZR patted the toy horse, looked at his mom, and exclaimed “啊 [a],” indicating a desire for the mom to lift him on the horse. Seeing his mom did not respond, YZR exclaimed “啊 [a]! 啊 [a],” turning to the research assistant ZYA, extending his hand to the latter at the same time. (YZR 1;01.10)
2. In a scene where the mom was holding the participant child YZX by the window, YZX stretched his neck to look at an airplane flying by outside the window, turned to look at the person recording the video, and pointed to the window, exclaiming: “啊 [a].” (YZX 1;02.06)

With a detailed examination of the aforementioned contextual interpretative elements, we can observe the co-occurrence frequency and proportion of different intonation words with intent interaction frameworks, communicative roles, and non-verbal multimodal elements. This allows us to gain insights into the circumstances under which intonation words express specific intentions.

## Results

### Data and data analysis

The data on the production of intonation words are presented in Table 2.

**Table 2:** Co-occurrence quantity and percentage of intonation words and contextual interpretation elements of the five children.

Contextual interpretation elements	Intent interaction framework			Communicative roles						Total	Non-verbal multimodal elements		
Frequency & percentage	Declarative	Interrogative	Imperative	Declarative		Interrogative		Imperative			Action	Facial Expression	Pointing
				Informant	Recipient	Questioner	Respondent	Command Giver	Command Receiver				
Intonation word													
啊/哎/欸 [ɑ/æ/ε]	150	15	186	144	5	2	13	167	20	351	243	82	57
	43%	4%	53%	97%	3%	13%	87%	89%	11%	43%	64%	21%	15%
嗯/呃/eng [ǝn/ǝ/ǝŋ]	79	48	233	58	21	7	41	175	58	360	355	88	76
	22%	13%	65%	73%	27%	15%	85%	75%	25%	45%	68%	17%	15%
咦[i]	0	1	2	0	0	0	1	0	2	3	3	0	0
	0%	33%	67%	/	/	0%	100%	0%	100%	0%	100%	0%	0%
哦 [oʊ]	27	60	9	22	5	0	60	1	8	96	67	18	10
	28%	63%	9%	81%	19%	0%	100%	11%	89%	12%	71%	18%	11%
Total count	256	124	430	224	31	9	115	341	88	810	668	188	143
	32%	15%	53%	88%	12%	7%	93%	79%	21%	100%	67%	19%	14%

**Figure 1:**
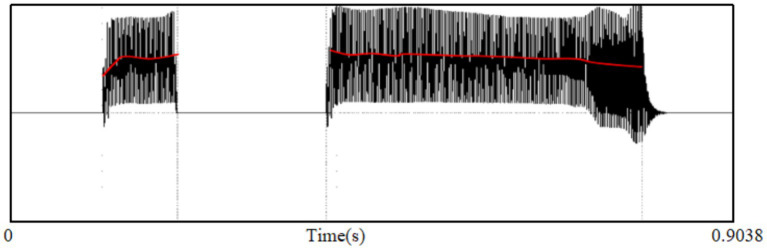
The pitch patterns of the two “啊 [a]”.

Furthermore, we use a pie chart to visually display the distribution proportions of intonation words in different intention interaction frameworks and communication roles. Please refer below to Figure 1.

Based on Table 2 and Figure 1, the following acquisition features are observed.

**Figure 2:**
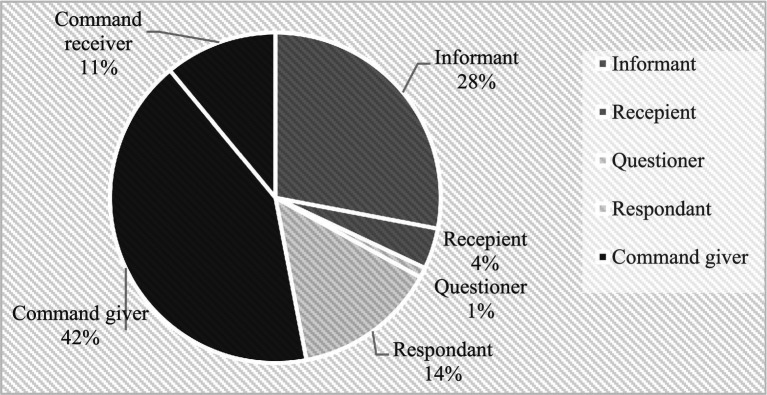
Distribution proportions of intonation words in different intention interaction frameworks and communication roles.

Different intonation words exhibit varying frequencies of use. Among them “啊 [a], 哎 [æ], 欸 [ε]” and “嗯 [ən], 呃 [ə], eng [əŋ]” are the most frequently used, applicable to all three intention interaction frameworks. “咦 [i]” shows the lowest usage, sporadically appearing in imperative and interrogative frameworks. “哦 [o]” is also produced, but its quantity is significantly lower than that of “啊 [a], 哎 [æ], 欸 [ε]” and “嗯 [ən], 呃 [ə], eng [əŋ].” In terms of the compatibility of intonation words with intention frameworks, the use of “哦 [o]” is relatively rare in imperative frameworks, but mostly employed in responses in interrogative frameworks and informative roles in declarative frameworks. “啊 [a], 哎 [æ], 欸 [ε]” and “嗯 [ən], 呃 [ə], eng [əŋ]” are used in all intent interaction frameworks, with the highest proportion in imperative frameworks and the lowest in interrogative frameworks. For instance, “啊[a]” yielded a statistically significant result (*F* = 64.809, *p* = 0.015; the between-group df is 6, and the within-group df is 2), indicating a notable difference (Figure 2).

Significant variation exists in communication intent across different intent interaction frameworks, with imperative functions prioritized. Imperative scenes have the earliest and highest production, while interrogative scenes, especially those with the child as the questioner, yield minimal production. Communication roles within these frameworks are unevenly distributed: children tend to be the imperative communicators in imperative frameworks and predominantly assume the role of the informant in declarative frameworks. In interrogative frameworks, the role of the respondent is dominant, with the child as the questioner constituting only 7% of instances. Non-verbal multimodal means are extensively used in intentional communication scenes, with actions being the most common, followed by facial expressions (such as eye movement), and pointing being the least used. Co-occurrence relationships between different non-verbal multimodal means and intent interaction frameworks are depicted in Table 3.

**Table 3:** Co-occurrence data of various non-linguistic multimodal means in different intent interaction frameworks for children.

Intent frameworks	Imperative framework		Declarative framework		Interrogative framework	
Frequency & percentage	Frequency	Percentage	Frequency	Percentage	Frequency	Percentage
Non-verbal multimodal means						
Action	374	69%	202	70%	92	54%
Expression	96	18%	24	8%	68	40%
Pointing	70	13%	63	22%	10	6%
Total	540	100%	289	100%	170	100%

Due to the highest output in imperative intent communication scenes and the lowest output in interrogative intent communication scenes, it can be predicted that the total output of the three non-verbal multimodal means will decrease sequentially from the imperative framework to the declarative framework, and then to the interrogative framework. However, based on the usage percentage of different non-verbal multimodal means in the same intent framework, some numerical variations can be observed: the proportion of action use has overwhelming advantages in all three intent interaction frameworks. However, in the declarative framework, the proportion of pointing use is slightly higher, 9 percentage points more than the imperative framework, and 16 percentage points more than the interrogative framework. This variation is meaningful. In declarative intent communication scenes, where the majority of the children’s communication roles are leading informants, children often express themselves by pointing with their fingers. Expressions are often not used independently, appearing either together with action or with pointing. Still, in the declarative framework, they are used the least, and in the interrogative framework, their co-occurrence with action has the highest frequency and proportion.

The above four aspects of acquisition characteristics indicate that the communicative intentions of children’s intonation words are very diverse. The specific interpretation of communicative intentions depends not only on the intent interaction framework and communication roles in the communication scene but also on non-verbal multimodal means. Non-verbal multimodal means are essential contextual conditions for the fine-grained interpretation of communicative intentions conveyed by intonation words. In terms of the output frequency in intent interaction frameworks, the high-frequency output in the imperative framework indicates that the communicative motivation of pre-linguistic children is significantly influenced by the functional needs of daily life. The uneven distribution of communication roles in different intent frameworks indicates that children tend to assume the leading role for conversational turns in communication exchanges. As for the preference for using “啊 [a], 哎 [æ], 欸 [ε]” and “嗯 [ən], 呃 [ə], eng [əŋ],” it may be related to the pronunciation features of these two groups of intonation words, which will be further analyzed below.

### Comparison of data for different children

We will now compare the intentional communication scenarios of the five participant children to identify commonalities and differences in communication contexts and frequencies. The specific data is presented in Table 4.

**Table 4:** Co-occurrence quantity and proportion of different children’s intonation words with various elements of contextual interpretation.

Contextual interpretation elements	Intent interaction framework			Communicative roles						Non-verbal multimodal elements		
Frequency & percentage	Declarative	Interrogative	Imperative	Declarative		Interrogative		Imperative		Action	Facial expression	Pointing
				Informant	Recipient	Questioner	Respondent	Command giver	Command receiver			
Participant children												
JBS	101	45	106	93	8	2	43	79	27	222	63	30
	40%	18%	42%	92%	8%	4%	96%	75%	25%	70%	20%	10%
LCY	92	53	93	80	12	5	48	73	19	181	54	47
	39%	22%	39%	87%	13%	9%	91%	79%	21%	65%	19%	16%
ZRY	25	10	54	18	6	2	8	44	12	77	35	22
	27%	11%	62%	75%	25%	20%	80%	79%	21%	58%	26%	16%
YZR	13	12	72	12	1	0	12	58	14	83	13	14
	14%	12%	74%	92%	8%	0%	100%	81%	19%	75%	12%	13%
YZX	25	4	105	21	4	0	4	89	16	105	23	30
	19%	3%	78%	84%	16%	0%	100%	85%	15%	66%	15%	19%
Total	256	124	430	224	31	9	115	343	88	668	188	142
	32%	15%	53%	88%	12%	7%	93%	80%	20%	67%	19%	14%

Based on Table 4, despite the difference in the absolute output values for intentional interaction frameworks, communication roles, and non-verbal multimodal means among the five children, the conclusion drawn regarding the initial use of intonation words and non-verbal multimodal means for intentional communication as well as the acquisition characteristics mentioned in the previous section remain valid. Of course, differences can be observed in the output of the five children, which are primarily manifested in two aspects: First, the frequency of intentional communication varies among the five children. JBS and LCY have higher communication frequencies, corresponding to higher usage frequencies of intonation words and non-verbal multimodal means. Second, in the intentional communication scenes of JBS and LCY, although the imperative framework is still the most frequent, the proportion of output in the declarative framework is significantly higher than the other three children. The output proportion of the interrogative framework is also slightly higher than the other three children. In addition, despite the differences in the types of intentions, communication roles, and the use of intonation words between the twin boys, the differences are not significant, and therefore, these insignificant distinctions can be disregarded.

Comparing the acquisition of intonation words in different children is meaningful. Through careful observation of each child’s video footage and discussions with their parents, it was discovered that JBS and LCY are more willing to collaborate with others, often informing about their discoveries and answering questions in intentional communication. LCY’s video footage began at 10 months, where multiple instances involved LCY using a combination of pointing and intonation words to inform others about what he saw, as well as using pointing or shaking heads along with intonation words to respond to others’ questions. This behavior was significantly different from the initial communication intentions of the twin boys and ZRY, which were predominantly imperative. JBS and LCY began producing holophrases at an earlier age, with LCY sporadically producing them at 1;02 years and JBS at 1;03 years, even though the pronunciation was not accurate (as shown in the examples (2) and (3) above with “给 [kε^55^]” and “不 [pǝ55]”). By 1;05 years, apart from kinship terms, they could articulate common nouns, verbs, and negations such as “袜袜 (socks),” “宝宝 (baby),” “gōugōu (referring to rooster),” “汪汪 (dog barking sound, referring to dog),” “表 (watch),” “灯 (lamp),” “姐姐 (sister),” “鸟 (bird),” “车 (car) or 滴滴 (car horn, referring to car),” “果果 (fruit),” “拿 (to take),” “来 (to come),” “要 (to want),” “给 (to give),” “没 (not have),” and “不 (not).” When intentional communication involved words that children could express, they often produced holophrastic utterances instead of using intonation words. In contrast, the other three participant children, by the data cut-off age (1;05 years for the twins and 1;04 years for ZRY), had not produced holophrases beyond kinship terms like “妈妈 (mom)” and “爸爸 (dad).” In conclusion, the earlier the production of holophrases, the higher the frequency of intonation word production, indicating a close correlation between the usage of intonation words and the time and quantity of holophrase production.

## Discussion

### Functional characteristics of intonation words

In previous studies, intonation words produced by children were often referred to as interjections, and sometimes as emotional vocalizations. However, such classifications may be inadequate. While intonation words can indeed serve as interjection substitutes in certain contexts (Xu and Tang, 1988: 393–483; Poggi, 2009; Liu, 2011), such as “咦 [i]” and “嗯 [ən],” which can function as expressions of questioning or affirmation, their functionality extends very much beyond this. Furthermore, intonation words do not necessarily convey the core functions of exclamations, such as expressing emotion and attitude (Zhao, 1926; Lü, 1982: 316; Guo, 2002: 236–237). In other words, considering intonation words as interjections does not fully encompass all the functions of intonation words, hence intonation words should be viewed as a more comprehensive term, including exclamatory functions and more. If we consider intonation words as holophrases or as components leading to holophrases, interjections with the function to serve as sentences fall under the category of holophrases. The logic of this is sound.

During the first weeks after birth, infants initiate vocalizations characterized as coos and murmurs (Oller, 2000) that elicit emotional and motivated responses from social partners. These vocalizations occur when infants are alert, relaxed, or playful. They are often accompanied by a focused, furrowed-brow gaze directed at a social partner, along with mouth movements resembling those of speech, known as pre-speech movements (Trevarthen, 1993). Both caregivers and inexperienced observers interpret these vocalizations as intentionally produced, purposeful, and requiring effort (Bloom and Lo, 1990; Beaumont and Bloom, 1993). Infants are known to engage in conversation-like exchanges from the end of the second month after birth. These ‘protoconversations’ involve both turn-taking and overlapping vocalization (Gratier et al., 2015). Alternatively, according to the Age-Appropriate Speech and Language Milestones proposed by NIDCD, children between 6 and 10 months of age begin to try to communicate by actions or gestures, while their vocal abilities continue to develop, such as babbling (“ba-ba-ba”) and attempting to repeat adults’ sounds. Some studies have found that children start using gestures to express requests, etc., as early as, 8 or 9 months old (Capone and McGregor, 2004; Goldin-Meadow, 2015; Mastrogiuseppe et al., 2015). According to the aforementioned studies, children’s babbling, vocalizations of emotion, or vocalizations with communicative intent precede gestures with communicative intent.

Based on this, we can consider whether intonation words can be seen as transitional elements from gestural actions to vocalized language components (such as holophrases). However, if this were the case, children should have a period of intentional communication solely through gestures before the production of intonational words. Then, there should be a stage where both intonational words and gestures are used together, leading quickly to the production of holophrases. But according to the above-mentioned research, children’s vocal intent communication occurs much earlier than gestures. Vocal intent communication is used independently for a period of time, while gestural actions are accompanied by vocal components from the beginning and cannot be used independently. The two are not easily separable. Furthermore, in nearly 100 h of video recordings, there is no evidence of gestural actions being used independently. This may be related to the difficulty in establishing joint attention with pure gestural actions and the difficulty in achieving communicative intent. Most of the time, when children engaged in intentional communication through gestures, they simultaneously produced “嗯 [ən],” or “啊 [a]” sounds.

Based on the observed facts and the acquisition data shown above, we consider intonation words as elements resembling holophrases or even as holophrases themselves. Their specific communicative intent requires interpretation within the interactive context. In contrast to holophrases, intonational words, though lacking lexical meaning in isolation, essentially fulfill a function akin to that of holophrases.

We consider intonational words as akin to holophrases or as holophrases based on the following two facts:

1. Although holophrases have tangible lexical meanings when viewed in isolation, the intent conveyed by children when producing independent words depends entirely on the interpretive elements in the interactive context. This is essentially no different from intonation words. While intonation words may lack inherent lexical meanings when viewed in isolation, in specific intent communication scenes, children’s non-verbal multimodal means can complement the intent indicated by intonation words. Let us examine Example (6):

(6) In a scenario where the elder sister and LCY were taking various toys from a corner of the couch, they took out the small hat belonging to the subject child. The elder sister asked: “Whose hat is this? Is it Pipi’s? Come on, put it on.” When the elder sister attempted to put the hat on LCY, the child twisted backward, uttering “不 [pǝ55],” then turned around, looking at the elder sister and lifting the arm, making an “哎 [æ]” sound. The elder sister said, “Oh, you want Sister to wear it. Alright, I’ll put it on.” She then wore the small hat on her own head, looking quite amusing. The elder sister asked LCY: “Does it look good?” LCY tilted his head back and laughed joyfully. LCY’s mother remarked: “Look, he is lost in entertainment again. He’s been having lots of moments like this lately.” (LCY 1;03.05)

In this communication scenario, the child used both holophrases and intonation words simultaneously. If the child, as the one being requested to wear the hat, responds with an intonation word like “嗯 [ən]” or “eng” instead of a holophrase, we can still understand the child’s intention not to wear the hat. Similarly, when the child, as the one requesting the sister to wear the hat, uses a holophrase like “戴 (to wear),” we still need to rely on the child’s communicative role and gestures in the context to understand the child’s intention for the sister to wear the hat. Without gestures, it is not clear whether the child is informing or requesting, and even if we understand it as a request, we may not know whom the child wants to wear the hat, the mother or the sister. Thus, in this intentional communication scenario in Example (6), there is no difference in the communicative intention and expressive function between intonation words and holophrases.

1. Children’s practical usage of intonation words for communication. Through detailed observations of communication in video recordings, we can conclude that intonation words, unlike interjections, do not express emotions and attitudes. Instead, they function similarly to nominal holophrases, to refer, inform, or command, just as they do in verbal holophrases, to inform or command. Occasionally, they also function similarly to negative holophrases, expressing negation. However, whether they function as a noun, verb, or negation depends on the intent interaction framework and non-verbal multimodal means for interpretation. For instance, in Example (7), LCY’s utterance “哎 [æ]” can be understood contextually as a nominal holophrase “表 (clock),” indicating a statement and informing others that “that is a clock” or “the clock is there.” Of course, it can also be interpreted more simply as a reference to the clock. The specific interpretation is not dictated by the intonation word itself, as the interpretation of the specific meaning of a holophrase is also inherently uncertain. In example (6), LCY’s utterance “哎 [æ]” can be interpreted contextually as a verbal holophrase “戴 (to wear),” indicating a command, meaning “sister, wear.” In Example (3), LCY’s utterance “欸 [æ]” accompanied by a head shake, can be interpreted as the negation word “不 (no),” meaning “not reading (but continue playing with the toy car).”

(7) LCY’s mother took him to ZRY’s house to play. Because it wasn’t his own home, LCY looked around. Suddenly, LCY pointed at the wall with his index finger and simultaneously uttered “哎 [æ].” LCY’s mother responded to the child, saying, “Oh, that’s a clock.” Then she said to everyone, “Pipi told you that it’s a clock. I just taught him at home yesterday.” At this moment, LCY pointed at the clock on the wall again, looked at the camera, and made the “哎 [æ].” sound once more. (LCY 0;10.22)

### Importance of intonation words acquisition

Tomasello (2008: 225) posits that the earliest use of communication conventions involves expressing complex concepts through holophrastic utterances, which reflect both reference and motivation (intention). Hence, “from a functional point of view, even holophrases are inherently composite, which might be seen as a kind of initial wedge into grammar.” they can be considered as the earliest rudiments of grammar. Given the functional equivalence of intonation words to holophrases, Tomasello’s (2008: 225) assertion regarding the pivotal role of holophrases in early syntactic development can be extended to intonation words. In other words, intonation words in children’s language hold a significant linguistic status. The acquisition of intonation words signifies the budding of early syntactic development, marking a milestone in the early development of syntax.

### The universality of intonation word acquisition

According to existing literature, the production of sounds like “嗯 [ən]” and “啊 [a]” before or simultaneously with holophrastic utterances is prevalent in cross-linguistic child language acquisition. This suggests that intonation words may be an indispensable path in children’s syntactic acquisition. Bornstein et al. (2004) and Soderstrom et al. (2008) found that English-speaking children also produce exclamations like “ah,” “oh,” and “eh” during the holophrastic phase, expressing imperatives or making statements. A close examination of naturalistic production data for a Japanese-speaking child, Nanami, before the age of 1;05, revealed the consistent production of intonation words with explicit communicative intent, such as あ/あっ [ɑ], うん [un], ん [n], え/えっ [ε], and so forth. Of course, examining the intonation words produced by children in Mandarin, English, and Japanese reveals both shared characteristics and distinctions in pronunciation features. A comparative analysis is presented in Table 5.

**Table 5:** Comparison of pronunciation features of intonation words produced by children of Mandarin, English, and Japanese.

	Intonation word	Pronunciation	Intonation word	Pronunciation	Intonation word	Pronunciation	Intonation word	Pronunciation	Intonation word	Pronunciation
Mandarin	啊/哎	ɑ/æ	欸	ε	哦	oʊ	嗯	ǝn	呃/eng	ǝ/ǝŋ
English	ah	ɑ/ɑː	eh	ε/eI	oh	oʊ				
Japanese	あ/あっ	ɑ	え/えっ	ε			うん/ん	un/n		

Table 5 shows both the commonalities and differences in the pronunciation features of intonation words produced by children in three languages. Commonalities include the use of [ɑ] and [ε] sounds, which are prevalent among children in all three languages. Additionally, the [oʊ] sound and the pronunciation with a nasal ending are used by children in at least two of the languages. Differences arise from variations in actual pronunciation, such as the English “ah” also being pronounced as the long vowel [ɑ:], although Mandarin-speaking children often pronounce “啊 [a]” as a long vowel as well, but since Mandarin does not use vowel length to distinguish meanings, it is not typically transcribed as a long vowel [ɑ:]. Moreover, Japanese-speaking children never used the diphthong [oʊ], and there is no scholarly mention of English-speaking children producing the monophthong with a nasal ending. Both English-and Japanese-speaking children have not been observed to produce the “呃/eng” used by Mandarin-speaking children. The differences in intonation word production among children in these languages are likely influenced by the phonetic characteristics of their respective native languages, that is the pronunciation features of children’s intonation words are influenced by adult input. Despite those differences, it can still be observed that the sound of children’s intonation words consists of phonemes that are the most natural and easiest for humans to produce. They are typically monophthongs or monophthongs with a nasal ending, which can be produced with the most natural tongue position and mouth shape. The inclusion of a nasal ending may be due to the natural resonance created as airflow enters the nasal cavity when the oral opening is not extensive. This choice of producing intonation words with the simplest and most natural sound in a specific context reflects both the constraints of early childhood articulatory abilities and suggests that if the sounds of children’s intonation words are phonemes that are the easiest for humans to produce, they may exhibit cross-linguistic universality.

### Syntactic initiation

The question that is more of interest here is why intonation words are considered a necessary step in syntactic acquisition. Specifically, why do children initiate syntactic learning with intonation words? The explanations provided earlier regarding infant vocalization abilities and the ease or difficulty of intonation word pronunciation suggest that the acquisition of intonation words may have cross-linguistic universality. This underscores the crucial role of intonation, a significant prosodic feature, in syntactic development. There is a reason to speculate that syntactic learning may be initiated by the rhythmic factor of intonation. Just like the linear structures preceding intonation, sentence-final intonation holds essential syntactic significance, as every sentence necessitates intonation. Initially, children are unable to produce complex phonemic units. Therefore, they attach intonation to the simplest and most phonetically feasible sound units, thereby initiating the rudiments of syntax. The concept of syntactic initiation has different connotations. The term “initiation” is used in this paper to refer to the mechanism through which children acquire syntactic knowledge. Many studies have explored the initiation of syntactic acquisition concerning prosody, such as that by Shi et al. (1999) and Werker and Vouloumanos (2000), and many more. The study by Shi et al. (1999), based on perceptual research, found that infants and toddlers, before the age of two, can interpret pseudo-words in experimental speech strings as nouns, verbs, and related syntactic structures based on prosodic cues and functional elements (such as articles). In other words, early children can engage in syntactic self-inference based on the interaction of functional morphemes and prosodic features. While both discussions focus on the initiation of syntactic acquisition, we view intonation as the initiation of children’s syntax differently from the study by Shi et al. (1999) in terms of conceptual implications. Of course, this does not necessarily imply contradiction.

## Other discussion

The extensive examination of intentional communication scenes involving five Mandarin-speaking children before the age of 1;05 revealed the production of a small but high-frequency set of intonation words such as “啊 [a]/哎 [æ]/欸 [ε],” “嗯 [ən]/呃 [ə]/ eng [əŋ],” “哦 [o],” and “咦 [i].” These intonation words, utilized within imperative, declarative, and interrogative frameworks, employed non-verbal multimodal means such as pointing, gesturing, and facial expressions to convey or receive commands, provide or receive information, and inquire or respond. By comparing the features of intonation words with those of holophrases in intentional communication, we believe that the function of intonation words is practically equivalent to that of holophrases. Therefore, the acquisition of intonation words signifies the beginning of syntactic acquisition. Based on the cross-linguistic universality of intonation word acquisition and its inherited relationship with sound symbolism before the production of intentional communication, this study proposes that syntax in children is initiated by the prosodic feature of intonation.

The most widely discussed idea proposed by Herrmann et al. (2012) and Tomasello (2012) is based on the characteristics of early individual communication relying on pointing gestures and the fact that chimpanzees can use some intentional gestures for communication. They put forth a bold hypothesis that human language originated from pointing and gesturing. However, the logical puzzle in this theory is: How did the transition occur from visual forms like pointing and gesturing to vocal language? Moreover, humans could entirely depend on complex gestural languages (such as modern sign languages) instead of spoken language to express intricate concepts, rendering the need for a transition to vocal language unclear.

A close examination of infants’ initial means of expression shows that vocalization for communicative purposes appears between 9 to 12 months, preceding intentional communication. For instance, infants produce vocalizations to convey a change in intention, such as urgent sounds when desiring to be fed. In terms of expression, the difference between these intention-altering vocalizations and intonation words lies in the latter being intentional communication based on joint attention cognitive skills, involving events or objects beyond the communicators themselves. In light of this, human individual’s vocal language did not originate solely from spontaneous gesturing. Also, gesturing and intonation words did not emerge sequentially; they appeared almost simultaneously. The early intonation words, possessing features of proxy words and syllabic boundaries, naturally extended from infants’ cooing and babbling, emotional vocalizations, or intention-altering vocalizations. Their emergence was facilitated by the prosodic characteristics of intonation and bootstrapped by the foundational cognitive skills of joint attention at opportune moments, driven by communicative functional needs. Infants’ vocal language and intentional gesturing seem to lack an evident lineage, hence it cannot be inferred that the emergence of vocalized language in human communities has a necessary evolutionary connection with chimpanzees’ intentional gestures or postures. Non-verbal multimodal means heavily dependent on interactive contexts have always coexisted with vocalized language. These forms of communication can complement each other, with more reliance on non-verbal multimodal means during the initial communication period when vocalization capabilities are not fully developed. Once children undergo approximately one and a half to 2 years of pronunciation training, their pronunciation abilities gradually mature with the emergence of joint attention and pattern-finding cognitive skills. They will begin to speak like adults, relying less or even ceasing to rely on gestures and body language.

## Data availability statement

The original contributions presented in the study are included in the article/supplementary material, further inquiries can be directed to the corresponding author.

## Ethics statement

The studies involving humans were approved by Ethics Committee of Capital Normal University. The studies were conducted in accordance with the local legislation and institutional requirements. Written informed consent for participation in this study was provided by the participants’ legal guardians/next of kin. Written informed consent was obtained from the individual(s), and minor(s)’ legal guardian/next of kin, for the publication of any potentially identifiable images or data included in this article.

## Author contributions

YuZ: Conceptualization, Formal analysis, Funding acquisition, Writing – review & editing. JL: Data curation, Investigation, Methodology, Writing – original draft. YaZ: Data curation, Investigation, Validation, Writing – review & editing.

## Footnotes

^1^The left [a] in the pitch diagram is a syllable with a higher pitch but a shorter duration, rising approximately 19 Hz within 90 ms (+208.8 Hz/s). The significant upward slope is sufficient for the listener to perceptually interpret the intonation as interrogative, functionally representing a questioning tone. The [a] on the right is a longer-duration syllable, descending about 105 Hz within 360 ms (−292 Hz/s), and the sudden drop at the end (−798.5 Hz/s) allows listeners to perceptually categorize the intonation as declarative, functionally representing a statement. While intonation patterns at the end of a sentence are complex, as discussed by Lin (2012), for monosyllabic intonation patterns in this study, we may disregard the intricate relations between intonation and syllabic tones, to simplify the analysis by focusing on the actual amplitude of rise and fall to determine whether it is rising or falling intonation.

^2^The characteristics of such emotional vocalizations vary widely among individual children, making it challenging to establish clear boundaries, and their nuances are difficult to capture in written descriptions. For instance, one of the participants, ZRY, frequently employs “bo-bo-bo-bo” to express joy and satisfaction, while using rapid and urgent vocalizations like “eng-eng-eng-eng” to convey a sense of urgency.
